# Force Degradation of Intermaxillary Latex Elastics: Comparative In Vitro and In Vivo Study

**DOI:** 10.1002/cre2.70072

**Published:** 2025-01-31

**Authors:** Lucie Ptáčková, Barbora Ličková, Wanda Urbanová, David Sluka, Klaudia Portašíková, Soňa Chamlarová, Iva Voborná, Ivana Dubovská

**Affiliations:** ^1^ Institute of Dentistry and Oral Sciences, Faculty of Medicine and Dentistry Palacký University Palackého Olomouc Czech Republic; ^2^ Department of Stomatology 3rd Medical Faculty Charles University and University Hospital Královské Vinohrady Prague Czech Republic; ^3^ Medical Faculty Charles University Pilsen Pilsen Czech Republic

**Keywords:** force degradation, initial force, intermaxillary elastics

## Abstract

**Objective:**

This study aimed to compare the force degradation of intermaxillary elastics (IE) in vitro and in vivo while stretching the IE to a precise diameter.

**Materials and Methods:**

IE 3/16″ medium Dentaurum from five different batches of packaging were analyzed. The in vivo study involved 10 volunteers, of which 100 IE were examined. To achieve three times the original diameter of the elastic, the distance between the upper canine and the lower dental arch was measured. Buttons were then placed in the mouth accordingly, and IE and passive aligners were inserted for five sessions of 48 h each. To investigate in vitro, 100 IE were placed in an incubator set at 37°C in a humid environment and stretched three times their diameter. The force of the elastics was measured in both investigations using a force meter at 0, 2, 8, 24, and 48 h.

**Results:**

In all patients except one, the three times diameter distance extended from the upper canine to the lower second premolar. The force degradation in vivo at 2, 8, 24, and 48 h was 20.58%, 26.78%, 34.81%, and 38.56% and in vitro was 16.38%, 22.83%, 28.32%, and 30.78%.

**Conclusions:**

The amount of stretching of IE varies for each patient when using standard insertion points. The force of IE decreases exponentially, the force degradation in vivo being higher. The clinician must consider the force decrease when advising the patient of the time interval to change the elastics.

## Summary

- The three times diameter distance corresponded to the distance between the upper canine and the lower second premolar in all but one patient.
- The force of the intermaxillary elastics decreased exponentially.
- The decrease in force was more pronounced in vivo than in vitro.
- The clinician must consider the force decrease when advising the patient of the time interval to change the elastics.

## 1 Introduction

Intermaxillary elastics (IE) are an integral part of orthodontic treatment with fixed appliances or clear aligners (Topouzelis and Palaska 2009; Thurzo et al. 2022). While inserted in the patient's mouth, they generate a force of designated magnitude and direction. Properties of IE should include flexibility, low costs, and a capacity for returning to their original form. Orthodontic elastics are made of natural latex or synthetic elastomer based on polyurethane. As synthetic non‐latex IE are not the focus of this research, they will not be mentioned any further. Natural latex is chemically *cis*‐1,4‐polyisoprene, an unsaturated hydrocarbon originating from the Brazilian rubber tree (*Hevea brasiliensis*), whose structure and molecular weight may vary depending on the plant species, region, or season (Wong 1976). The flexibility and strength are improved during a process called vulcanization—cross‐linking occurs in the presence of sulfur and other compounds (Bokobza 2018; Perrella and Gaspari 2002). As natural latex is very sensitive to ozone stabilizers, antioxidants and anti‐ozone agents are further added during the production of IE to give the latex the desired properties (Sambataro et al. 2019). During the manufacture of the IE, steel rods of varying widths are dipped into the vat of the material—the more times the dipping occurs, the thicker the latex layer and therefore the resulting latex tubing will be. The tubes are then cut to the desired width, resulting in elastic rings of defined diameter and thickness. Depending on the width and thickness of the latex layer, the IE are then divided by manufacturers into various sizes and “light,” “medium,” and “heavy” according to the exerting force (Sambataro et al. 2019).

It is a well‐known fact that the amount of force exerted by the elastics decreases over time as every elastomeric material undergoes creep and stress relaxation (Santos et al. 2009). Plastic deformation of the polymer under load unravels cross‐links leading to mechanical degradation together with chemical degradation leading to the degrading of the mechanical properties. Furthermore, if large, excessive forces are used, the chains may slip on each other, resulting in the permanent deformation of the material. Natural latex is very sensitive to ozone, solar radiation, ultraviolet radiation, or free radical‐producing systems—unsaturated double bonds are broken at the molecular level and the polymer chain is weakened. After the individual pieces of IE are produced, the ozone‐permeable surface is increased and bond breakage occurs more rapidly—latex elastics should not be used after the expiry date precisely because of their reduced durability (Wong 1976).

The force degradation of orthodontic latex IE is the highest after initial elongation and then decreases gradually during the following hours. However, the rate of the force decrease differs widely between the studies—the decrease in force within the first 24 h ranges from 14.2% to 90.2% (Yang et al. 2020; Oliveira et al. 2017). The rate of force degradation differs between manufacturers, different sizes and strengths of the IE (Oliveira et al. 2017; Dubovská et al. 2023; Wang et al. 2007; Qodcieh et al. 2017), and the conditions in which the IE is stored between the measurements (Kardach et al. 2019). The degradation of force can also be influenced by the design of the study although not many studies studied the force degradation of elastics while worn by the patient (Yang et al. 2020; Wang et al. 2007; Qodcieh et al. 2017; Pithon et al. 2016).

This study aimed to compare the force degradation of one specific type of latex IE in vitro in a controlled humid environment and in vivo in patients' mouths stretched to the precise diameter.

## 2 Methods

In vivo and in vitro measurements of force degradations of IE were performed. Based on our previous research, the 3/16″ medium Dentaurum (Dentaurum, Ispringen, Germany) IE were selected for investigation as they had the closest initial force to the declared force of 1.255 N when prestretched and stretched to three times diameter (Wang et al. 2007). According to the Safety Data Sheet of Dentaurum GmbH & Co. KG, these elastics are made of Natural rubber (Caoutchouc) together with Sulfur, Zinc Oxide, Age Resistor, and Vulcanization Accelerator (Dentaurum GmbH & Co. 2016, 2021). Ethical approval for the study was obtained from the local ethics committee. The study was conducted following the Declaration of Helsinki and current local legal regulations.

Two hundred pieces of elastics 3/16″ medium from Dentaurum were analyzed from five different batches of packaging 20 pieces each in vivo and in vitro. A total of 1000 measurements were made. All elastics were within their use‐by date, delivered by the manufacturer no later than 2 weeks before the measurement, manufactured no later than 2 months before the measurement, and, after being received from the manufacturer, stored in sealed plastic containers in a dark environment. All elastics were subjected to “prestretching” immediately before time 0 measurement— they were stretched to three times the original diameter, according to the recommendations by Proffit and Liu (Proffit et al. 2019; Liu, Wataha, and Craig 1993).

**Figure 1:**
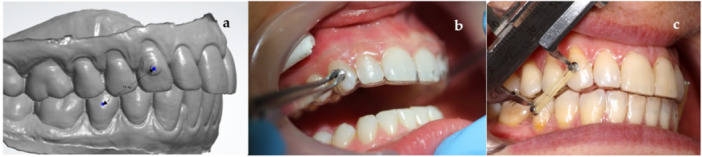
Preparations for in vivo study. (a) 3D‐printed model of the participant's teeth with the measured distance, (b) button insertion according to an aligner template made on a 3D‐printed model, and (c) a participant with IE and passive aligners in place.

For an in vivo examination, 10 volunteers were recruited and written informed consent was obtained from all the participants. Every participant acquired 10 IE 3/16″ medium from Dentaurum (Dentaurum, Ispringen, Germany) and wore one pair for 2 days while coming in for the measurements at a given interval. None of them were undergoing orthodontic treatment, all were Angle Class 1 in the first permanent molars. Beforehand dental scans were taken in all of them using the 3Shape scanner, and virtual models were created (Figure 1a). On the virtual models, the distance from the upper canine to the lower dental arch was measured to achieve exact distances equaling three times the diameter of the 3/16″ elastics—that is, 14.4 mm. An aligner template made on a 3D‐printed model was used to accurately glue orthodontic buttons. The buttons were glued in the patient's mouth with an orthodontic adhesive in a standard manner according to the premade template and the distance between the two buttons was re‐measured (Figure 1b). Passive stabilizing aligners were fabricated on the 3D‐printed model and inserted into the participant's mouth (Figure 1c). The study participants were instructed on how to properly remove and insert the aligners and IE. They were also advised to remove both before eating or drinking and keep their appliance removals to a minimum. To maintain the study's accuracy, the participants were asked to continue their normal eating habits.

**Figure 2:**
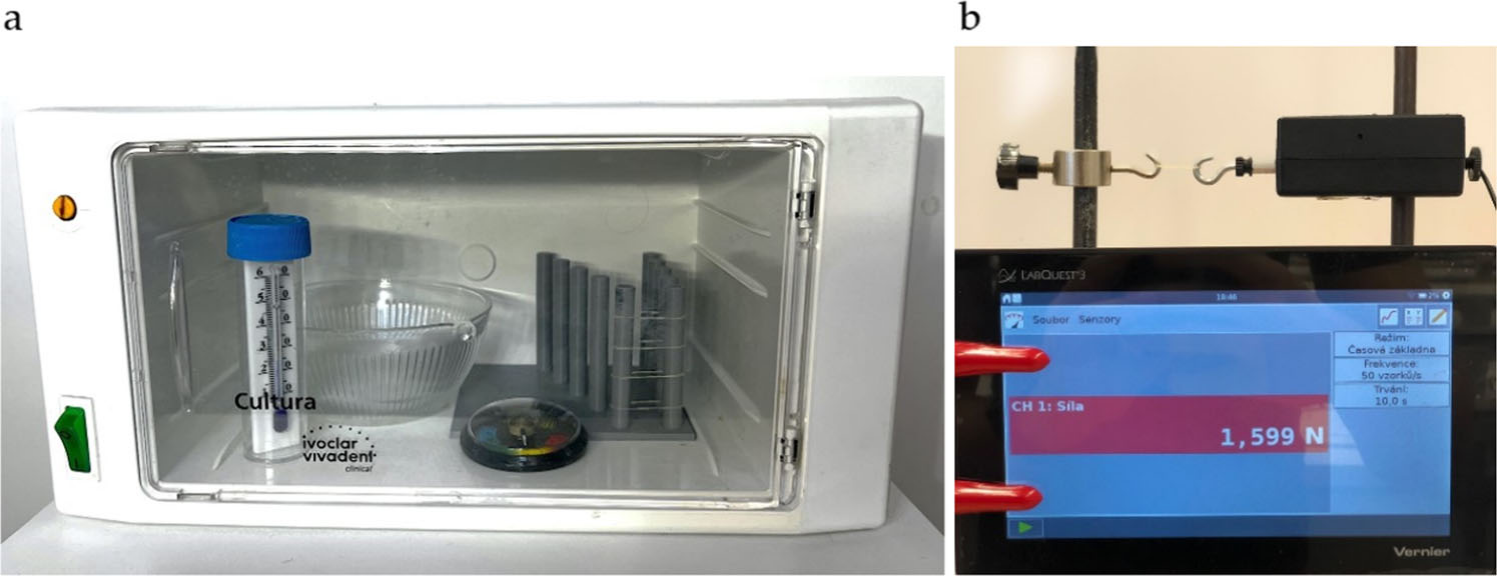
(a) An incubator with a thermometer, humid meter, and 3D model of the board with spurs for standardization of IE stretching. (b) Elastic stretched to the exact distance on the force meter “ScienceCube” connected to the portable data logger “LabQuest3.”

After prestretching, each IE force was measured one by one at time 0 before the first insertion into the participant's mouth with a force meter from the company “ScienceCube” set on the exact distance, calibrated before each set of measurements. The force meter was connected to the portable data logger “LabQuest3” from the Vernier company (Figure 2b). The right elastic was always inserted first followed by left elastic 10 min afterward. Elastics were measured five times: at time 0, at 2, 8, 24, and 48 h. Patients came 10 min before the time limit and elastics were measured directly after removal from the oral cavity and were inserted right back. Each patient has worn 10 elastics for 48 h (5 pairs for 2 days each). After the measurements were completed, the buttons were removed from the participant's mouth and residual orthodontic adhesive was cleared in a standard manner.

For in vitro examination, 100 IE 3/16″ medium from Dentaurum (Dentaurum, Ispringen, Germany) were measured five times: at time 0, at 2, 8, 24, and 48 h. To standardize the stretching conditions in vitro between measurements, a 3D model of a board with spurs was created in the “Rhinoceros 3D” program and subsequently printed using the “Prusa i3 MKS+” printer. The distance between spurs corresponded with the three times diameters of IE from Dentaurum = 14.4 mm. The bulk of the five elastics were subjected to the experiment each time. Simulation of the oral environment in the laboratory was made possible using the Ivoclar Vivadent Cultura incubator at a constant temperature of 37°C and a controlled humid environment, where the stretched elastics were stored between the measurements (Figure 2a). The conditions in the incubator were continuously monitored using a precision thermometer and a humidity sensor. The force of elastics was measured individually with a force meter from the company “ScienceCube” set on the exact distance, calibrated before each set of measurements. The force meter was connected to the portable data logger “LabQuest3” from the Vernier company. The measurements were repeated after 2, 8, 24, and 48 h and results were recorded in newtons.

Statistical processing of the collected data from both examinations was performed on the statistic software IBM SPSS Statistics for Windows, Version 23.0., IBM Corp., Armonk, NY. The collected data were analyzed by Shapiro–Wilk normality tests which showed normal force distribution. Further statistical processing was performed using parametric methods which were validated with nonparametric tests. The use of parametric methods is appropriate given the relatively large range of samples. Data were presented using means and standard deviations. A comparison of two independent sets was performed using a two‐sample *t*‐test.

## 3 Results

Shapiro–Wilk normality tests showed that the distribution of forces is normal for most parameters. Parametric methods were used for processing and validated by non‐parametric tests. The use of parametric methods is appropriate due to the relatively large range of samples. A total of 1000 measurements were made in each group in vivo and in vitro. Data are presented using means and standard deviations in Table 1. A comparison of two independent sets was performed using a two‐sample *t*‐test. All tests were performed at a significance level of 0.05, if the *p* value was less than 0.05.

**Table 1:** Force degradation progress for in vivo and in vitro measurements.

	Hours	In vivo (*n* = 100)				In vitro (*n* = 100)				
Force degradation		Mean	SD	Min	Max	Mean	SD	Min	Max	*p*
Force in time (N)	0	1.31	0.14	1.06	1.84	1.30	0.22	0.86	1.99	0.790
	2	1.03	0.12	0.72	1.37	1.08	0.18	0.60	1.41	0.019*
	8	0.95	0.12	0.64	1.39	1.00	0.16	0.58	1.35	0.025*
	24	0.85	0.13	0.38	1.22	0.92	0.14	0.61	1.28	0.0002***
	48	0.80	0.11	0.45	1.14	0.89	0.12	0.62	1.18	< 0.0001***
Decrease of force during time (%)	2	20.58	9.96	−1.89	45.11	16.38	7.82	−2.15	38.14	0.001***
	8	26.78	8.93	0.00	50.00	22.83	8.37	1.94	44.76	0.001***
	24	34.81	8.86	11.71	66.67	28.32	7.73	9.71	47.10	< 0.0001***
	48	38.56	7.69	21.62	67.15	30.78	7.91	11.83	47.74	< 0.0001***
Average decrease (%) per 1 h	2	10.29	4.98	−0.94	22.56	8.19	3.91	−1.08	19.07	0.001***
	8	1.03	1.59	−2.66	4.40	1.08	0.77	−2.10	3.08	0.818
	24	0.50	0.55	−1.13	1.82	0.34	0.24	−0.39	0.91	0.009*
	48	0.16	0.23	−0.38	1.04	0.10	0.13	−0.24	0.48	0.046*

Abbreviations: max, maximal force; mean, mean force; min, minimal force; N, Newton, SD, standard deviation; *p*, *p* value.

*p < 0.05, ***p < 0.001.

At time 0, there was no statistically significant difference between IE for in vivo and in vitro. At all other times, the force was statistically significantly higher in the in vitro mode. At 2 h, the in vitro force was 1.08 N and the in vivo force was 1.03 N. At 4 h, the difference in force was 0.05 N; in vitro force was 1 N, and in vivo force was 0.95 N. At 24 h, the difference in force was 0.07 N and at 48 h it was 0.09 N. With a longer time, the residual force difference was higher for IE in vitro than for elastics used in vivo. The force degradation was significantly higher for IE in vivo. In the first 2 h, there was a 20.58% decrease in force in vivo and only 16.38% in vitro, the force degradation being the greatest. The decrease in force averaged over this time interval was 10.29%/h for elastics in vivo and 8.19%/h for elastics in vitro.

Over the next 8 h, the force decreased by 26.78% in vivo and 22.83% in vitro. After 24 h, there was a decrease in force of 34.81% for in vivo and 28.32% for in vitro. After 48 h, there was a decrease of 38.56% for in vivo and 30.78% for in vitro. The force degradation after the first 2 h was significantly reduced—below 1.08%/h.

**Figure 3:**
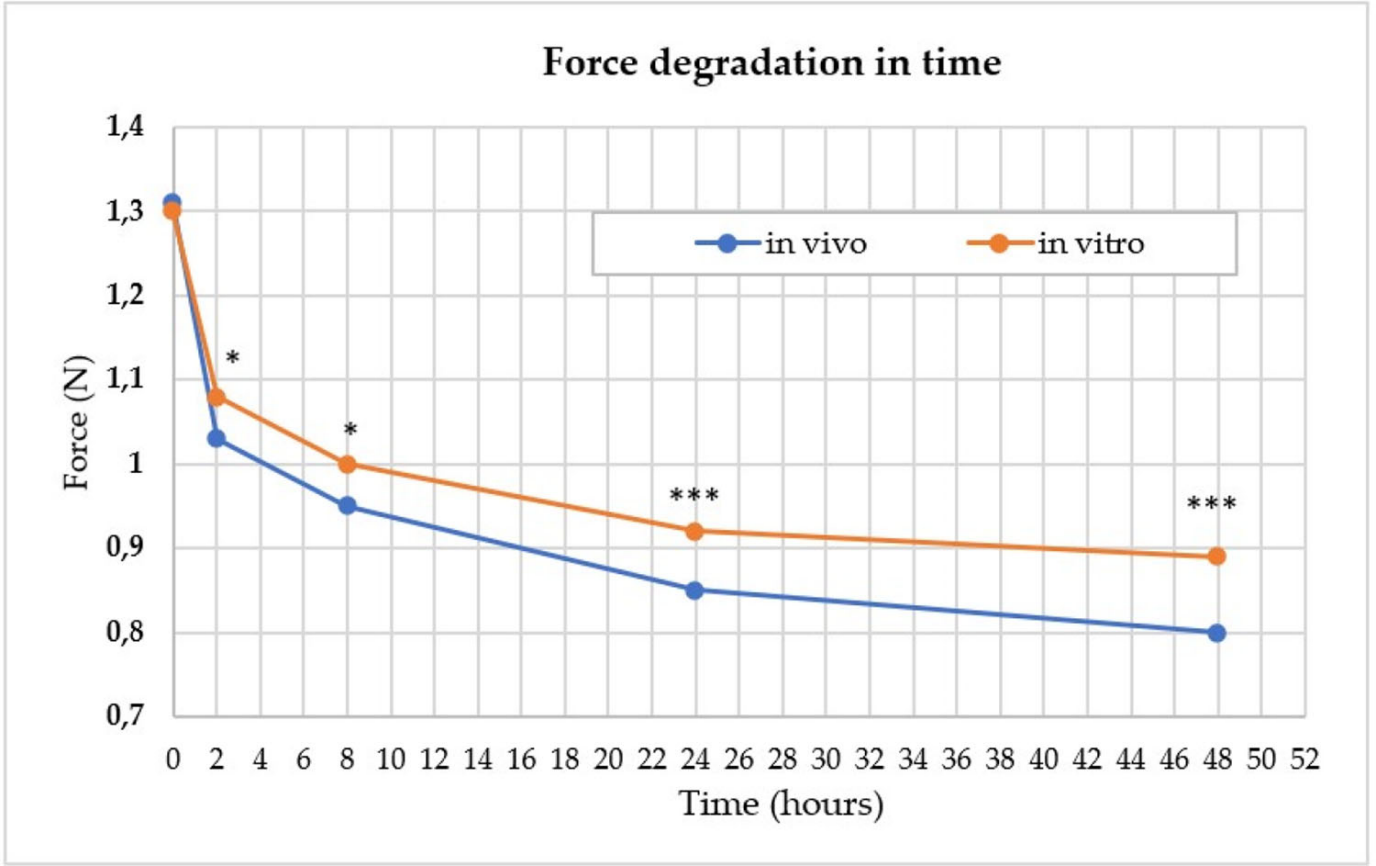
Graphic representation of the force degradation in time in vivo and in vitro.

At time 0 h, the differences were not statistically significant. At all other times, the force is statistically significantly higher in the in vitro setting (Figure 3).

The measurement error was checked using Dahlberg's formula (*D*). Since the calculation of the Dahlberg error does not account for the magnitude of the measured values, it is more appropriate to use the relative Dahlberg error (RDE), which is obtained by dividing the Dahlberg error by the average of the corresponding measured values, to compare the accuracy of the measurements for individual parameters. After multiplying by 100, it is given as a percentage. The degree of absolute agreement between the first and control measurements was verified by calculating the intraclass correlation coefficient (ICC). The occurrence of systematic error was verified by paired *t*‐test.

**Figure 4:**
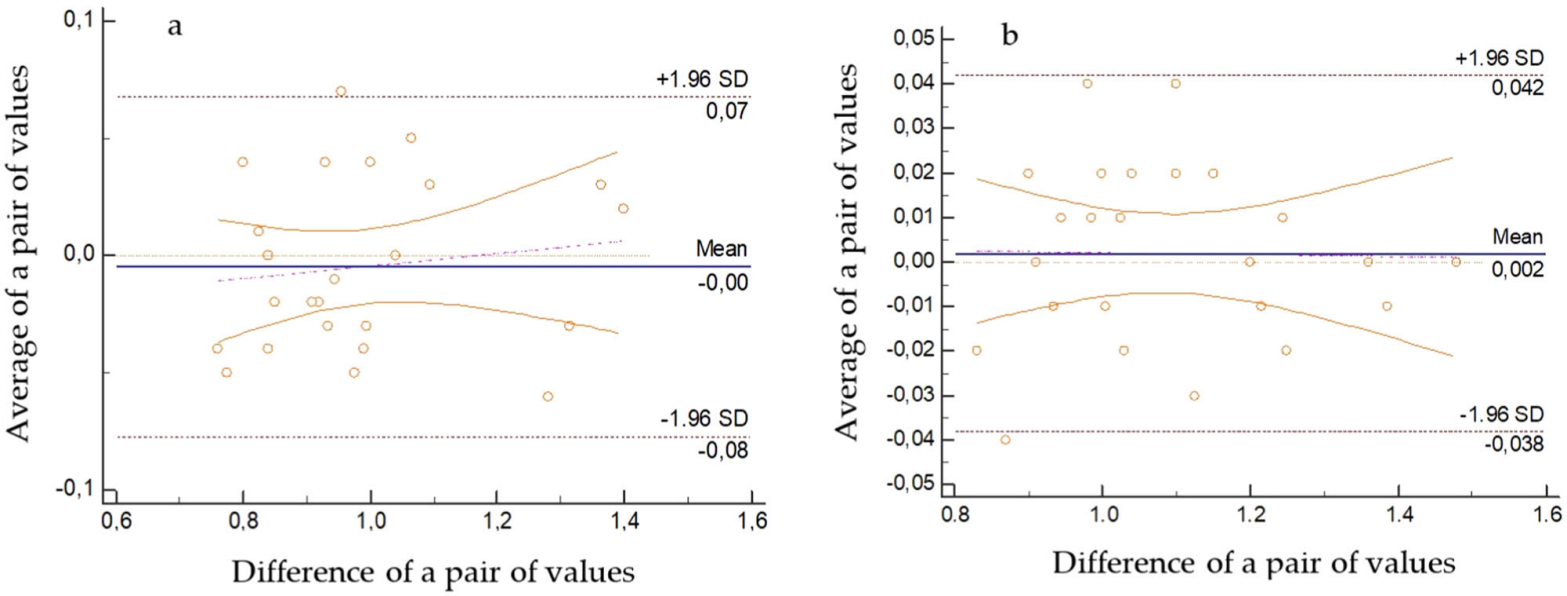
Graphic representation of Bland–Altmann plots comparing the results of the first and second control measurements (a) in vivo and (b) in vitro. Orange dashed line—horizontal zero line; blue line—average error; pink dashed line—regression line indicating the trend in the error rate; orange curves—95% confidence interval bands; brown dashed line—limits of agreement are given by the bands; value of the mean standard deviation ±1.96 SD in which 95% of the measurements should lie.

The RDE was 2.59% for in vivo measurements and 1.29% for in vitro measurements. The higher measurement error for the in vivo study setting is probably due to the more complex measurement after extraction of the IE from the patient's mouth than from the 3D model and therefore risks a longer delay. The ICC values were 0.980 in vivo and 0.993 in vitro, representing a perfect match (Table 2). If the ICC values are greater than 0.75 and the RDE is less than 8%, the measurement is considered sufficiently accurate (Proffit et al. 2019). The paired *t*‐test revealed no systematic error (*p* values were greater than 0.05). Bland–Altman plots were used to graphically represent the error (Figure 4A,B). These graphs are used to reveal the possible dependence of measurement errors on the magnitude of the measured value. Therefore, we can conclude that our measurements are sufficiently accurate, the errors are random and there are no significant trends.

**Table 2:** Error measurements.

Measurements setting	*D*	RDE	ICC	*p*
In vivo	0.026	2.59%	0.980	0.549
In vitro	0.014	1.29%	0.993	0.822

Abbreviations: *D,* Dahlberg error; ICC, intraclass correlation coefficient; *p, p* value of paired *t*‐test; RDE, relative Dahlberg error.

In all participants but one, the distance three times the diameter of the IE extended from the upper canine to the lower second premolar; in one participant, the distance corresponded to the standard insertion points—the upper canine and the lower first molar.

## Discussion

Various studies have been conducted on the force degradation of IE. However, there is inconsistency in the results due to the use of different types of elastics and experimental methods, which makes it difficult to compare the findings (Young 1977). In the present study, we used elastics from one manufacturer, Dentaurum (Dentaurum, Ispringen, Germany) size of 3/16″, and a strength of “medium.” The physical properties of these elastics were examined in two environments: in vivo and in vitro, when stretched three times their diameter. These elastics were chosen because, in preliminary in vitro study their initial force was closest to the value declared by the manufacturer (Dubovská et al. 2023).

The present study found that the greatest force decrease in the strength of IE was observed in the first 2 h of use, which confirms the results of previous studies (Liu, Wataha, and Craig 1993; Kanchana and Godfrey 2000a; Barrie and Spence 1974). However, some researchers have reported the greatest force decrease after 24 h, suggesting that the elastics should be replaced once a day in clinical practice (Oliveira et al. 2017; Pithon et al. 2016). Our findings contradict this recommendation, as we observed that the force decrease between 0 and 24 h was around one‐third of their initial force, and the force decrease was the lowest between 24 and 48 h in the monitored period. This is consistent with other studies that found that the force remained relatively constant for a few days after the 24 h force degradation (Wang et al. 2007; Beattie and Monaghan 2004; Lopes Nitrini et al. 2019; Gangurde, Hazarey, and Vadgaonkar 2013; Andreasen and Bishara 1970). Liu, Wataha, and Craig (1993) in their research concluded that the force of IE remained almost stable after 1 day because the structural changes in the elastomer caused by repeated stretching were not cumulative (Liu, Wataha, and Craig 1993). While the force of IE decreases at a slow rate after 1 day, mechanical damage to the elastics occurs after several hours of use in a patient's mouth (Wang et al. 2007; Gurdán et al. 2022). Therefore, regular replacement of elastics is essential to maintain their initial force. Some authors recommend replacing them every 6 h, while others suggest intervals of 12 h (Castroflorio et al. 2022; Gioka et al. 2006). Our results suggest that replacing the IE every 8 h might be advisable to maintain the level of force between 75% and 100% all the time.

It is important to consider various factors when evaluating the force decrease of IE, including the time aspect, environmental factors such as salivary alkalinity, temperature changes, stretching repetition and intensity (Liu, Wataha, and Craig 1993; Beattie and Monaghan 2004; Kanchana and Godfrey 2000b; Hwang and Cha 2003). Storing intermaxillary elastic packages in an unsuitable environment could alter the elastomer structure and affect their properties (Wong 1976). It is important to use, for study purposes, IE within the expiry date and properly store and utilize IE from different batches, which was not always followed in previous studies. This is consistent with the general characteristics of elastomer degradation, where temperature, fluids, chemicals, and UV radiation can degrade the elastomeric structure. Saliva and bacteria can infiltrate the molecular structures on the latex rubber surface, resulting in discoloration and expansion (Kanchana and Godfrey 2000b; Brantley et al. 1979). Therefore, the medium in which elastics are tested and the study design should greatly affect the force decrease. Only two studies from the 1980s did not find any differences in force decrease for different environments (Andreasen and Bishara 1970). Later studies found that force degradation was higher in distilled water after 8 and 24 h than in dry air conditions (Qodcieh et al. 2017; López et al. 2012). Wong (1976) stated that greater force degradation was observed in wet conditions than in dry conditions of the same temperature. Many authors have used artificial saliva as a suitable medium, with the force degradation being around 25% within 24 h (Yang et al. 2020; Wang et al. 2007; Kardach et al. 2019). However, the composition of artificial saliva solutions varies throughout the studies, which may account for the differences in the results while using this medium. Interestingly, in the study of Oliveira et al. (2017), where one batch of stretched elastics was stored at room temperature in artificial saliva, degradation of slightly less than 10% in 24 h compared to a dry environment was shown, raising the question whether it is the higher temperature that has a greater effect on degradation than the saliva itself (Perrella and Gaspari 2002). There are not many studies focusing on force degradation of the IE in clinical settings. Although the exact force decrease results differ, all concluded that force degradation in in vivo settings is higher (Yang et al. 2020; Wang et al. 2007; Qodcieh et al. 2017). Our study found that the IE worn by patients had a 5% higher mean force degradation in 48 h than when stored in an incubator at a constant temperature of 37°C and a controlled humid environment. Different conditions affect the force degradation of IE, so any future studies on the subject should be designed in vivo.

It is important to note that the magnitude and dynamics of stretching can have an impact on the results of force degradation (Castroflorio et al. 2022; Klabunde and Grünheid 2022). Manufacturers recommend that IE should be stretched three times their diameter to achieve the declared force. In our research, we found that when the stretching was measured to exactly three times the diameter, the insertion points were typically on the upper canine and lower second premolars for all patients except one. The same conclusion was also reached by Castroflorio et al. (2022). As the standard insertion points for orthodontic treatment with fixed appliances or aligners are the upper canine and lower first molars, it is clear that without measuring the force with a force meter in each clinical case, clinicians cannot be certain of the force generated by IE. Therefore, clinicians must bear in mind that simulated force degradation models may not be accurate in clinical situations.

Limitations of the study: It is important to note that there are some limitations to this study. The in vivo research was performed on 10 participants who wore 10 IE gradually (5 pairs for 2 days each). The intensity of force degradation may have been affected by individual differences in eating habits, oral parafunctions, and the duration of time that the elastics were inserted. Additionally, the in vitro investigation of the elastics was conducted in batches of 10 and measured gradually, resulting in a time difference between the first and last one, which could have influenced the results.

## Conclusion

The stretching distance of IE varies for each patient when using standard insertion points. As a result, the initial force may differ in each case. The force of IE decreases exponentially in both in vivo and in vitro settings, with the highest decrease in force occurring in the first 2 h. However, in vivo, the force degradation was higher by 5% on average, and the initial force dropped to three‐quarters after 8 h, gradually decreasing further. The clinician must consider the force decrease when advising the patient of the time interval for changing the elastics.

## Author Contributions

Conceptualization: Ivana Dubovská and Iva Voborná. Methodology: Ivana Dubovská. Validation: Soňa Chamlarová and Klaudia Portašíková. Investigation: Barbora Ličková, Lucie Ptáčková, Klaudia Portašíková, and David Sluka. Resources: Ivana Dubovská and Klaudia Portašíková. Data curation: Barbora Ličková, Lucie Ptáčková, David Sluka, and Klaudia Portašíková. Writing–original draft preparation: Barbora Ličková, Wanda Urbanová, and Ivana Dubovská. Writing–review and editing: Ivana Dubovská, Iva Voborná, Wanda Urbanová, and David Sluka. Visualization: David Sluka and Soňa Chamlarová. Supervision: Soňa Chamlarová and Iva Voborná. Project administration: Ivana Dubovská and Wanda Urbanová. Funding acquisition: Iva Voborná. All authors have read and agreed to the published version of the manuscript.

## Disclosure

The funders had no role in the design of the study; in the collection, analyses, or interpretation of data; in the writing of the manuscript; or in the decision to publish the results.

## Ethics Statement

Ethical approval for the study was obtained from the Ethics Committee of the University Hospital and the Faculty of Medicine Palacky University in Olomouc, Czech Republic (approval number: 136/23).

## Conflicts of Interest

The authors declare no conflicts of interest.

## Data Availability

The data that support the findings of this study are available from the corresponding author upon reasonable request.
